# AXL receptor tyrosine kinase: a possible therapeutic target in acute promyelocytic leukemia

**DOI:** 10.1186/s12885-021-08450-y

**Published:** 2021-06-17

**Authors:** Mariam Fatima, Salik Javed Kakar, Fazal Adnan, Khalid Khan, Afsar Ali Mian, Dilawar Khan

**Affiliations:** 1 Department of Healthcare Biotechnology, Atta-ur-Rahman School of Applied Biosciences, National University of Sciences and Technology, H-12, Campus, Islamabad, Pakistan; 2 Department of Industrial Biotechnology, Atta-ur-Rahman School of Applied Biosciences, National University of Sciences and Technology, Islamabad, Pakistan; 3 Integrated Chinese and Western Medicine Postdoctoral Research Station, Jinan University, Guangzhou, China; 4 Center for Regenerative Medicine and Stem Cell Research, The Aga Khan University, Karachi, Pakistan

**Keywords:** Acute promyelocytic leukemia, Retinoic acid receptor-α, PML/RARα, All-*trans* retinoic acid, Wnt/β-catenin pathway, AXL, Receptor tyrosine kinase

## Abstract

**Background:**

Acute promyelocytic leukemia (APL) is a subset of acute myeloid leukemia (AML) which is characterized by the fusion of promyelocytic leukemia PML and retinoic acid receptor- alpha (RAR-alpha) genes. All-trans retinoic acid (ATRA) and/or arsenic trioxide (ATO) have resulted in durable cytogenetic and molecular remissions in most APL patients and have altered the natural history of the disease. Most APL patients treated with ATRA and/or ATO are now anticipated to have a nearly normal life expectancy. Unfortunately, relapse and resistance to the current treatment occur in APL patients and the outcome remains dismal in these refractory patients.

AXL receptor tyrosine kinase (AXL-RTK) has been shown to increase tumour burden, provide resistance to therapy and is critical to maintain cancer stem cells (CSCs) in chronic myeloid leukemia (CML) by stabilizing β-catenin in the Wnt/β-catenin signalling pathway. However, the role of AXL-RTK has not been explored in PML/RARα-positive APL. This study aimed to explore the role of AXL-RTK receptor in PML/RARα-positive APL.

**Methods and results:**

By using biochemical and pharmacological approaches, here we report that targeting of AXL-RTK is related to the down-regulation of β-catenin target genes including *c-myc* (*p* < 0.001), *AXIN2* (*p* < 0.001), and *HIF1α* (*p* < 0.01) and induction of apoptosis in PML/RARα-positive APL cell line. Resistance to all-*trans* retinoic acid (ATRA) was also overcomed by targeting AXL-RTK with R428 in APL (*p* < 0.05).

**Conclusion:**

Our results provide clear evidence of the involvement of AXL-RTK in leukemogenic potential of PML/RARα-positive APL and suggest targeting of AXL-RTK in the treatment of therapy resistant APL patients.

**Supplementary Information:**

The online version contains supplementary material available at 10.1186/s12885-021-08450-y.

## Background

AML is a heterogeneous, most common type of acute leukemia [1] that involves mutation in hematopoietic and progenitors stem cells (HPSCs) leading to uncontrolled division, self-renewal and differentiation [2]. APL, a subtype of AML that constitutes 5–10% of all AML, which has hallmark chromosomal t (15;17)(q22;q12) translocation. This translocation leads to a formation of fusion gene which encodes leukemia associated fusion protein (LAFP) PML/RARα [3]. It is identified by the particular growth of immature myeloid precursors whose differentiation is blocked at the promyelocytic stage [3].

Currently, about 80% of APL patients achieve complete remission when all-*trans* retinoic acid (ATRA) and/or arsenic trioxide (ATO) are combined with conventional chemotherapy [4]. However, in spite of promising results, relapse and refractory to the treatment have been observed in the clinical setting. One of the important reasons of ATRA resistance is mutation in the ligand binding domain (LBD) of RARα which can be found in 40% of the relapse patients [5]. ATO therapy is effective comparatively [6] however, relapse remains a clinical reality. Resistance to ATO therapy is attributable to mutations in the B2-domain of PML/RARα which continues to pose a clinical challenge in APL patients [7]. Hence, non-specificity of chemotherapy, inability of ATRA and ATO as alone or in combination for APL therapy, and arsenic poisoning [8] impel to seek other treatment options of APL.

Tyrosine kinase inhibitors (TKIs) have sought great interest in recent years especially in the field of cancer research with promising results in clinical trials [9]. One of the receptor tyrosine kinases (RTKs), AXL, has become a novel target for leukemia therapy. AXL belongs to TAM family of RTKs, which includes TYRO3, AXL, and MER. Growth arrest-specific protein 6 (Gas6) ligand activates AXL-RTK [10], followed by activation of different signalling cascades that lead to cell survival, proliferation, migration and invasion through activation of Wnt/β-catenin, PI3K, MAPK, and JAK/STAT [11]. AXL-RTK is expressed in a relatively low levels throughout an adult body [12] and was first identified in patients with chronic myeloid leukemia (CML) [13]. However, anomalous AXL/Gas6 has been associated with tumour development, progression and reduced survival rate in number of malignancies, including breast cancer [14] non-small cell lung cancer [15] pancreatic cancer [16] oesophageal cancer [17] hippocampus neuron [18].

Bone marrow derived stem cells (BMDSCs) are educated by AML cells in order to release Gas6 that ultimately aids in proliferation of leukaemic cells and developing therapeutic resistance due to the activation of AXL-RTK [2]. AML patients exhibit high AXL expression after treatment with chemotherapy [19]. A variety of AXL-RTK inhibitors have been reported in literature [10] but the most specific inhibitor is R428, the first inhibitor to enter clinical trials in 2014 [20].

In light of the issues discussed above, new targets need to be explored to increase the treatment options for APL patients and to lower the risk of resistance developed from single and cotargeting. In the present work we investigated (i) expression pattern of AXL-RTK in NB4 cells (ii) role of AXL-RTK in the proliferation potential of NB4-sensitive and resistant cells (iii) role of AXL-RTK in the regulation of Wnt/β-catenin signalling in APL.

## Materials and methods

### Cell lines and chemicals

Three cell lines were used in this study: NB4, U937 and K562. All these lines were obtained from our lab stocks generated from the German Collection of Microorganisms and Cell Cultures (DSMZ, Braunschweig, Germany) vials. These cell lines were maintained in RPMI 1640 supplemented with 10% fetal calf serum (FCS; Gibco/Invitrogen, Karlsruhe, Germany), 1% L-glutamine and 1% penicillin/streptomycin (Gibco/Invitrogen, Karlsruhe, Germany). AXL-RTK inhibitor R428 was purchased from Med-Chem-Express, New Jersey, USA and dissolved in dimethyl sulfoxide (DMSO) (Sigma, Steinheim, Germany) to a 1000X stock solution, which were further diluted to 1X working concentrations for the experiments. ATRA was purchased from Sigma Aldrich, and dissolved in absolute ethanol according to manufacturer protocol. 

#### Cell viability test

The cell growth and viability were assessed by counting cells using haemocytometer with dye exclusion (dead cells) as described previously [21] before MTT assay.

### In vitro cytotoxicity assay

The effect of different inhibitors on cellular proliferation of U937, K562 and NB4 cells was measured using MTT cell proliferation assay kit (Roche, Basel, Switzerland), according to the manufacturer’s instructions. Briefly, 10,000 cells were plated in 96-well plates in the presence/absence of the inhibitors, either alone or in combinations, at various concentrations as listed in Table 1 for 72 h in their specified medium. For control, medium was added to the cells containing only vehicle. Next, 15 μL of filter-sterilized MTT (5 mg/mL in PBS) was added and the cells were incubated for 3–4 h at 37 °C in a CO_2_ incubator (Galaxy 170 R, New Brunswick) for crystal formation. The MTT medium was then removed carefully without disturbing the crystals and 150 μL DMSO was added to each well to dissolve the crystals. Cells were incubated for 15 min before measuring the absorbance. Absorbance was measured at 550 nm, and 660 nm was taken as reference wavelength using multiscan GO plate reader (PR 4100 Bio-Rad). For data analysis, the proliferation of untreated/control cells was taken as 1, and the proliferation in the treated cells was expressed relative to the untreated/control cells.

**Table 1:** Different concentrations of ATRA and R428 used

Inhibitor	Concentrations used (μM)		
ATRA	0.41	0.82	1.62
R428	0.62	1.25	2.50

### RNA extraction for gene expression studies

Two million cells were centrifuged at 1500 rpm for 5 min. Supernatant was removed and pellet was suspended in 1 mL of TRIzol reagent to extract total RNA (Life Technologies) according to standard protocols. RNA pellet was air dried at room temperature in the laminar flow hood. 20 μL of nuclease-free water was then added to suspend the RNA pellet. The quality of RNA was checked by 2% agarose gel electrophoresis by visual examining the quality of bands. The extracted RNA was quantified using Nanodrop 2000 (Thermo Fisher Scientific, USA) and purity was confirmed through 260/280 ratio before storing at − 80 °C until further processing.

### Complementary DNA synthesis

1 μg of RNA template was used for cDNA synthesis using cDNA Synthesis Kit (Solis BioDyne) according to the manufacturer’s protocol. cDNA quality was checked and primer optimization for *GAPDH* (housekeeping gene), *AXL*, *c-myc, AXIN2 and HIF1α* was undertaken by conventional PCR using standard protocols. The details of the primers for the expression of the above genes in U937, K562 and NB4 cells are described in Table 2.

**Table 2:** Primer properties for the amplification of genes of interest [22]

Gene/ Primer	Sequence	GC%	Annealing Temperature
*GAPDH*-Forward	CCTGCACCACCACTGCTTA	57.8%	59.9 °C
*GAPDH*-Reverse	CATGAGTCCTTCCACGATACCA	50.0%	59.5 °C
*AXL-Forward*	GTCGGACCACTGAAGCTACC	60.0%	60.1 °C
*AXL-Reverse*	CATCGTCTTCACAGCCACCT	55.0%	60 °C
*c-myc-Forward*	CAGCGACTCTGAGGAGGAAC	60.0%	59.8 °C
*c-myc-Reverse*	TCGGTTGTTGCTGATCTGTC	50.0%	58.2 °C
*AXIN2*-Forward	TCAAGTGCAAACTTTCGCCAACC	47.8%	62.8 °C
*AXIN2*-Reverse	TAGCCAGAACCTATGTGATAAGG	43.4%	57.0 °C
*HIF1α*-Forward	CAGATCTCGGCGAAGTAAAG	50.0%	60.4 °C
*HIF1α*-Reverse	TCACAGAGGCCTTATCAAGATG	45.5%	60.8 °C

### Gene expression analysis

Gene expression at transcriptional level was analysed by real-time polymerase chain reaction (qPCR; Applied Biosystems, Darmstadt, Germany 7300). The master mix for qPCR used was SYBR Green Master Mix (5X) (Solis BioDyne). The qPCR program consisted of an initial cycle of 50 °C for 02 min and 95 °C for 03 min followed by 40 cycles at 95 °C for 10 s and at 60 °C for 1 min. The details of the primers for the expression of the above genes in U937, K562 and Nb4 cells are described in Table 2 [22]. The Ct values were exported into a Microsoft Excel worksheet for calculation of fold changes according to the comparative Ct method. The expression of the target was normalized to endogenous GAPDH gene is given by 2^-ΔΔCt^ [23].

### Apoptosis analysis

The apoptosis was evaluated by DNA fragmentation assay. U937, K562 and NB4 cells were cultured in 6-well plate and treated with R428 and incubated for 72 h. After incubation, DNA was extracted as described by [24]. DNA pellet was air-dried for about 30 min, resuspended in 40 μL of nuclease-free water and quantified using Nanodrop 2000 (Thermo Fisher Scientific USA). DNA was equally loaded on 1.5% agarose gel and resolved using gel electrophoresis technique. The gel was examined in ultraviolet gel transilluminator [24].

### Statistical analysis

All experiments were performed technically and biologically in triplicates. Results were presented as mean +/− SD. The data was analysed using one-way ANOVA and student t-test and *p*-value less than 0.05 was considered as significant. Statistical analyses were carried out using GraphPad Prism5 (GraphPad Software Inc. San Diego, CA).

## Results

### AXL-RTK expression in NB4 cell line

**Fig. 1:**
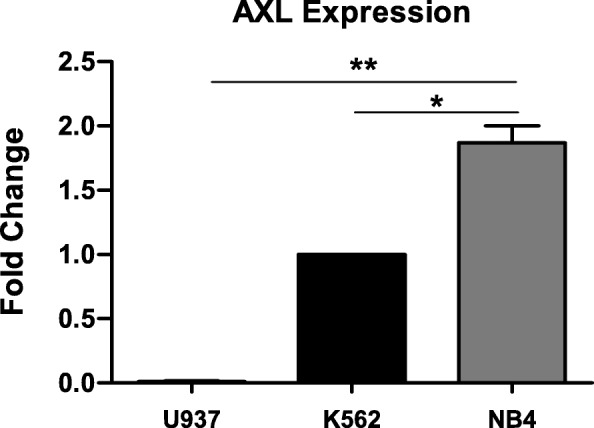
Expression analysis of AXL-RTK in U937, K562 and NB4 cell lines. Cells were cultured in liquid medium (RPMI 1640 + 10% FBS + 1% L-glutamate and 1% penicillin/streptomycin). Total RNA was extracted from U937, K562 and NB4 cells. The expression level of AXL-RTK was analysed using qPCR. The Ct values were normalized to that of GAPDH gene and results are represented as 2^-ΔΔCt^. Statistical significance was tested using student t-test (*p*-values < 0.05 are considered statistically significant). The means +/− SD of triplicates for one representative experiment out of three performed are given

AXL-RTK play a pivotal role in various cancers including leukemia [22]. Therefore we investigated the expression pattern of AXL-RTK in APL. As a leukemic model we used NB4, an APL cell line expressing PML/RARα, K562, a CML cell line expressing M-BCR-ABL1 as positive control and U937, an AML cell line as negative control for AXL-RTK expression at transcriptional level [25]. Expression pattern of AXL-RTK was assessed by using real-time PCR. As shown in the Fig. 1, NB4 cells showed the higher expression of AXL-RTK compared to K562. Whereas, no expression of AXL-RTK was observed in U937.

Taken together these data show strong expression of AXL-RTK in NB4 cell line.

### Pharmacological targeting of AXL-RTK strongly interferes with the proliferation potential of NB4 cell line

Expression of the AXL-RTK in NB4 cells prompted us to find whether there is a significant association between expression of AXL-RTK and the proliferation potential of NB4 cell line. To dissect the role of AXL-RTK in the proliferation of NB4 cells, U937, K562 and NB4 cells were treated with increasing concentration (0, 0.62, 1.25 and 2.50 μM) of a selective AXL inhibitor R428 [26]. U937 and K562 cells were used negative and positive control respectively. Proliferation/cytotoxicity was assessed using MTT assay. DMSO was used as vehicle control.

**Fig. 2:**
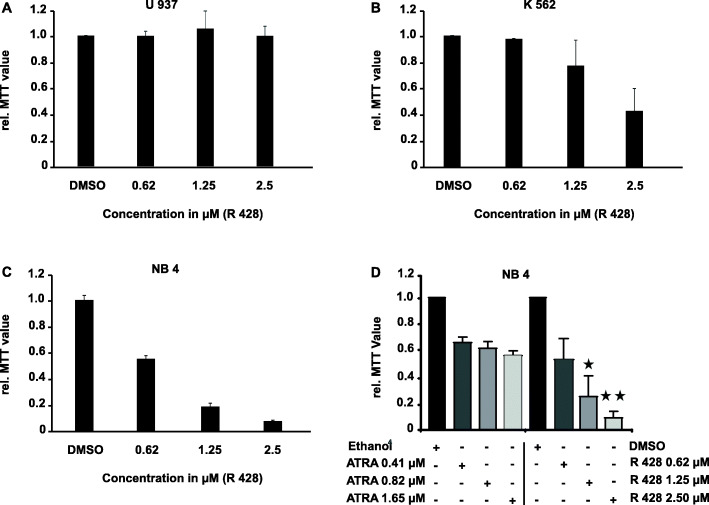
Effect of R428 and ATRA on the proliferation potential of PML/RARα-positive NB4 cells. Cells were cultured in RPMI 1640 medium + 10% FBS + 1% L-Glutamate and 1% Pencillin and Streptomycin. MTT assay was performed for (**A**) U937, (**B**) K562 and (**C**) NB4 cells upon exposure to 0.01% DMSO and indicated concentrations of R428 and ATRA. (**D**) Comparison of R428 and ATRA in the inhibition of NB4 cells after 72 h. The mean +/− SD of triplicates from one representative experiment out of three performed is given

We found that R428 inhibited cellular proliferation in a dose-dependent manner with an IC-50 of 0.7 μM in NB4 cells and 2.41 μM in K562 cells with no effect on the proliferation of U937 (Fig. 2).

As ATRA is independently inhibiting the growth of NB4 cells, we were interested to compare the effects of ATRA and R428 in the growth of NB4 cells. For this purpose, we treated the NB4 cells with increasing concentration of either ATRA or R428 as shown in (Fig. 2D) and performed MTT assay. As depicted in Fig. 2D, ATRA and R428 both were able to inhibit the growth of NB4 cell line with stronger effect of R428 on the growth of these cells.

To determine whether the inhibition of proliferation in NB4 cells by R428 is co-related with the induction of apoptosis, these cells were treated with increasing concentrations of R428. Apoptosis was assessed by DNA fragmentation assay after 72 h. We observed that increasing concentrations of R428 induced apoptosis in NB4 cells (Supplementary Figure 1).

Taken together our results show that proliferation of NB4 cells is AXL-RTK dependent.

### Inhibition of AXL-RTK with R428 and PML/RARα with ATRA showed additive anti-proliferative effect in NB4 cells

**Fig. 3:**
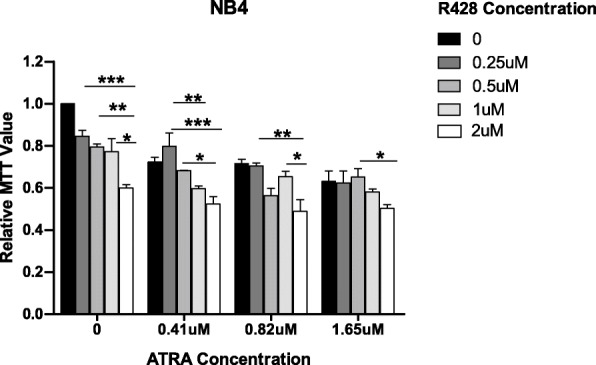
Effect of single (R428 or ATRA) and combine treatment (R428 + ATRA) on the proliferation potential of PML/RARα positive NB4 cells. Cells were cultured in RPMI medium + 10% FBS + 1% L-Glutamate and 1% Penicillin and Streptomycin to determine the proliferation potential of NB4 cells in the presence of 0.01% DMSO and indicated concentrations of R428 and ATRA. Cell proliferation was assessed through MTT assay after 72 h. (*p*-values < 0.05 are statistically significant). Bars show mean +/− SD

Our results show that AXL-RTK is up-regulated in NB4 cells and targeting AXL-RTK strongly interferes with the proliferation potential of NB4 cell line. We have also shown that ATRA is able to inhibit the growth of NB4 cells. Therefore, we went on to know whether combined treatment of NB4 cells with R428 and ATRA can further decrease the proliferation potential of NB4 cells in order to reduce the toxicity of ATRA. We treated NB4 cells with the indicated low concentration of ATRA and R428 either alone or in combination as shown in (Fig. 3) and performed MTT assay. From Fig. 3 it can be seen that single targeting with either ATRA or R428 reduce the proliferation of NB4 in a dose dependent manner while co-targeting of these cells using both the inhibitors showed anti proliferative effect in an additive manner.

In summary we can conclude that ATRA and R428 can be combined for efficient inhibition of NB4 cells.

### Inhibition of AXL-RTK with R428 can overcome resistance to ATRA

**Fig. 4:**
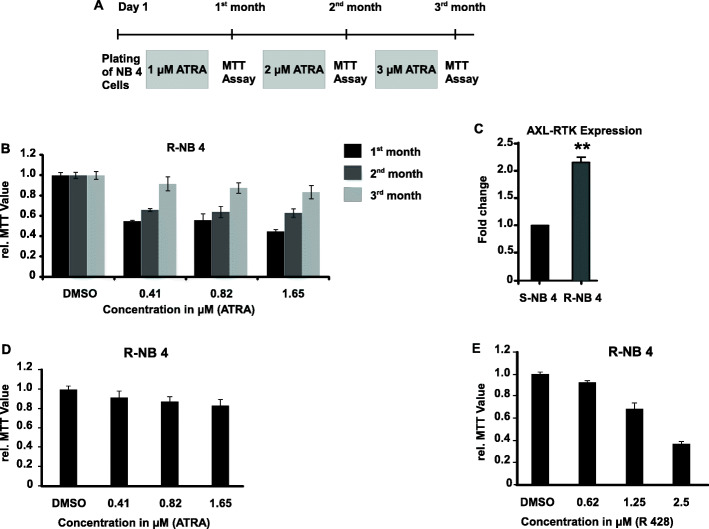
Effect of R428 on ATRA resistant NB4 cells. Cells were cultured in RPMI medium + 10% FBS + 1% L-Glutamate and 1% Penicillin and Streptomycin in the presence of DMSO, 1 μM, 2μΜ and 3 μM of ATRA for first, second and third months respectively (**A**) Methodology for development of ATRA resistant NB4 cell line. (**B**) MTT assay performed for the first, second and third month for development of ATRA resistant NB4 cell line in the presence of 0.01% DMSO and indicated concentrations of ATRA. (**C**) q-PCR for the expression of AXL-RTK in ATRA sensitive and resistant NB4 cells. (**D**) MTT assay for R-NB4 cells treated with indicated concentration of ATRA. (**E**) MTT assay for R-NB4 cells treated with indicated concentration of R428 (p-values < 0.05 are statistically significant). Bars show mean +/− SD

AXL-RTK is a key mediator of developing treatment resistance in various cancers including prostate, breast, ovarian, colorectal and lung cancers [27]. It has widely been shown that the patients with APL develop resistance to ATRA and ATO and treatment options for such patients are limited or unavailable [28, 29]. Therefore, we were interested to explore the role of AXL-RTK resistance in APL. For this purpose we first made NB4 cell line resistant to ATRA as described elsewhere [30] and shown in (Fig. 4A and B). NB4 cells were treated with ATRA for three months and each month MTT was performed in order to look for the development of resistance. ATRA resistance was seen by their sensitivity to ATRA. As shown in (Fig. 4B) as compared to the first month, cell population was increased in the second and third month. After confirmation that NB4 is resistant to ATRA and it is known that AXL-RTK provides resistance in various cancers, therefore we first looked for the expression of AXL-RTK in sensitive and resistance NB4 cells through real-time PCR. As shown in (Fig. 4C) AXL expression was increased in the resistant NB4 cell line as compared to the sensitive one. To confirm whether this resistance in proliferation is due to the upregulation of AXL-RTK, therefore, we performed MTT assay and treated the resistant NB4 cell line with either ATRA or R428 as shown in (Fig. 4D and E) respectively. From the (Fig. 4D) it can be seen that ATRA was unable to reduce the proliferation and overcome the resistance but molecular targeting of AXL-RTK with R428 interfered with the proliferation potential of resistance NB4 cells (Fig. 4E).

In summary we conclude that ATRA resistance can be overcome by targeting AXL-RTK with R428.

### Molecular targeting of AXL-RTK with R428 is accompanied by the inhibition of Wnt/β-catenin signalling

**Fig. 5:**
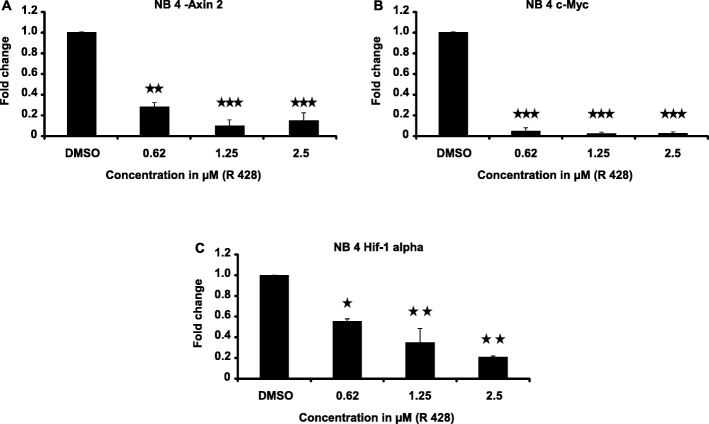
Effect of targeting AXL-RTK on the expression of Wnt/ β-catenin target genes. Cells were cultured in liquid medium (RPMI + 20% FBS + 1% L-Glutamate and 1% Pen/strep) in the presence of 0.01% DMSO and indicated concentrations of R428. Expression analysis was done through qPCR. (**A**) Expression pattern of *AXIN2*. (**B**). Expression pattern of *c-myc* (**C**). Expression pattern of *HIF1α*. (*p*-values < 0.05 are statistically significant). Bars show mean +/− SD

In order to understand the mechanism of inhibition of proliferation and whether AXL-RTK was also involved in activation of Wnt signalling in APL, we performed real-time PCR for Wnt target genes including *AXIN2* [31], *c-myc*, and *HIF1α* [32]. It can be seen in Fig. 5 that targeting AXL-RTK by R428 is able to affect β-catenin target genes in NB4 cells i.e. a significant down- regulation of *c-myc, AXIN2* and *HIF1α* was observed in treated cells as compared to the control cells. Hence it can be concluded from these findings that AXL-RTK is involved in the regulation of β-catenin in PML/RARα-positive APL.

In summary our results show that AXL-RTK is involved in the leukemogenic potential of PML/RARα in APL.

## Discussion

The present study aimed to explore the role of AXL-RTK as a potential therapeutic target in PML/RARα-positive APL, its role in developing resistance to current therapeutics and the mechanism underlying resistance and inhibition of proliferation in APL. We have been able to deduce that AXL-RTK was highly expressed in PML/RARα-positive NB4 cells. We found that R428 was able to interfere with the proliferation potential of PML/RARα-positive NB4 cells and can overcome resistance to ATRA. It was also deduced that the combined treatment (R428 + ATRA) had an additive effect on the proliferation of NB4 cells. Furthermore, the study was able to conclude that the antiproliferative effect of R428 was related to the induction of apoptosis and down regulation of Wnt target genes.

AXL-RTK has been shown to regulate Wnt signalling by stabilizing β-catenin and activate signalling pathways including PI3K/AKT/mTOR, Ras/Raf/MAPK/ERK [33]. Among all the members of TAM family, only AXL has been shown to be involved in the progression of various cancers including glioblastoma, breast cancer and CML [22, 34, 35]. Consistently we have seen high expression of AXL-RTK in NB4 cells as well. Over-expression of AXL has been shown to be involved in the stabilization and increased protein level of β-catenin in CML which is confirmed by the up-regulation of β-catenin target genes. Our findings indicate that the pharmacological targeting of AXL-RTK suppresses the proliferation of NB4 cells which is related to down-regulation of several Wnt/β-catenin target genes including *c-myc*, *AXIN2* and *HIF1α,* suggesting the role of AXL-RTK in the regulation of Wnt/β-catenin pathway in APL. As PML/RARα leukemogenesis is Wnt/β-catenin dependent and this pathway is the main pathway in regulating the self-renewal of CSCs [36], so further studies on the role of AXL-RTK in PML/RARα-positive CSCs can aid in eradicating CSCs to avoid relapse.

ATRA and ATO is the standard therapy for the treatment of APL. But there are problems of toxicity with standard therapy that is the induction of differentiation syndrome [37, 38]. Also drug resistance is the main challenge in the clinical management of cancer. In APL, resistance to ATRA and ATO may derive from the mutations in the RARα ligand binding domain (LBD) and in the PML-B2 domain of PML-RARα, but such mutations cannot explain the majority of resistances experienced in the clinic, globally accounting for 5–10% of cases. This suggests that there are other players which may account for the resistance. The role of AXL-RTK in developing resistance to therapies is well-known and well-studied in different cancers. AXL-RTK also promotes resistance to targeted cancer therapies. Several groups have reported that AXL is up-regulated in response to EGFR inhibition and the signalling pathway is mediated by AXL. Up-regulation of AXL gene expression at transcription level is found to be associated with developing resistance in cisplatin-resistant ovarian cancer cells [39], which is in accordance with our findings that ATRA-resistant NB4 cells have high AXL-RTK expression as compared to the ATRA-sensitive NB4 cells. Targeting AXL enhances the therapeutic efficacy of chemotherapy and other small-molecule inhibitors, such as VEGF, EGFR, PI3K, PARP, and HER2 inhibitors. We have shown that R428 has antiproliferative and additive antiproliferative effects and can overcome the therapeutic resistance to ATRA in PML/RARα-positive APL. This would be helpful for the therapeutic management in refractory APL. However, we explored the role of AXL in leukemic cell line which needs to be further validated in in vivo model and primary samples of APL patients and may pave the way in proposing an effective therapy for PML/RARα-positive APL in future.

## Conclusion

These results of our study explored the role of AXL-RTK in the biology of PML/RARα-positive APL and suggest a possible candidate target for therapeutic intervention in refractory APL.

## Supplementary Information

**Additional file 1.**

## Data Availability

All the data generated during this study are included in this article and as supplementary information.
