# Supplementary material for: AXL receptor tyrosine kinase: a possible therapeutic target in acute promyelocytic leukemia
Source: BMC Cancer. 2021 Jun 17;21:713. doi: 10.1186/s12885-021-08450-y (PMC8210361; doi:10.1186/s12885-021-08450-y)
Supplement: Supplementary file 1 — Additional file 1. [file 12885_2021_8450_MOESM1_ESM.pdf]

Title of paper

AXL Receptor Tyrosine Kinase: A Possible Therapeutic Target in Acute Promyelocytic Leukaemia

Author(s): Mariam Fatima[1], Salik Javed Kakar[1], Fazal Adnan[2], Khalid Khan[3], Afsar Ali Mian[4] and Dilawar Khan[1]*,

[1]Department of Healthcare Biotechnology, Atta-ur-Rahman School of Applied Biosciences,

National University of Sciences and Technology, Islamabad, Pakistan

[2]Department of Industrial Biotechnology, Atta-ur-Rahman School of Applied Biosciences,

National University of Sciences and Technology, Islamabad, Pakistan

[3]Integrated Chinese and Western Medicine Postdoctoral Research Station, Jinan University,

Guangzhou, China

[4]Center for Regenerative Medicine and Stem Cell Research, The Aga Khan University, Karachi,

Pakistan

***Correspondent Footnotes:**

Dilawar Khan, PhD

Department of Healthcare Biotechnology

Atta-u-Rahman School of Applied Biosciences

National University of Science and Technology

H-12, Campus, Islamabad, Pakistan

Phone: +92 (0) 51 9085 6139

Email: dkhan@asab.nust.edu.pk

dilawar_qau@yahoo.com

Supplementary Figure

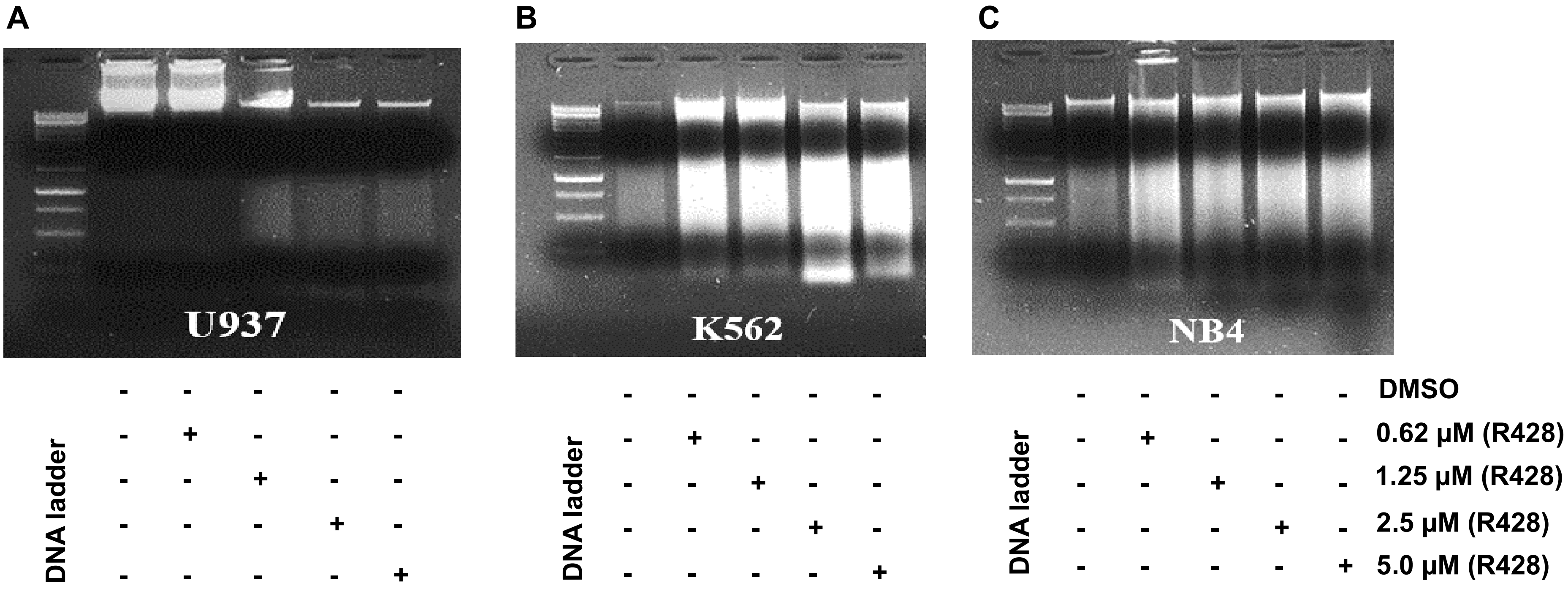

Supplementary figure. DNA fragmentation assay was utilized for apoptosis analysis. Cells U937 (A), K562 (B) and NB4 (C) were cultured in 6-well plate, treated with inhibitor R428 and incubated for 72 hrs. DNA was extracted and equal amount of DNA was loaded for each sample and gel electrophoresis was done on 1.5% agarose gel. The gel was examined on ultraviolet gel transilluminator.
